# Novel 3-(3, 5-difluoro-4-hydroxyphenyl)-1-(naphthalen-2-yl) prop-2-en-1-one as a potent inhibitor of MAP-kinase in HeLa cell lines and anti-angiogenic activity is mediated by HIF-1α in EAC animal model

**DOI:** 10.18632/oncotarget.27836

**Published:** 2020-12-15

**Authors:** Dileep Kumar M. Guruswamy, Kyathegowdana Doddi Srivinavasa Balaji, Kattepura Krishnappa Dharmappa, Shankar Jayarama

**Affiliations:** ^1^Department of Biotechnology, Teresian College, Siddhartha Nagara Mysore-570011, Karnataka, India; ^2^Department of Studies in Biochemistry, Mangalore University, Chikkaaluvara, Kodagu-571232, Karnataka, India; ^3^Department of Food Technology, Davanagere University, Karnataka-577002, India

**Keywords:** novel chalcone, Bcl2, caspase 3, BAX, receptor tyrosine kinase inhibitor

## Abstract

In the present investigation, we synthesized chalcone bearing naphthalene compound d1, and on the basis of ^1^H-NMR, ^13^C NMR, and LC-MS data we had specified the structure of the synthesized compound. The resultant compound d1 was assessed for their antiproliferative action against human cancer cell lines (HeLa, HCT116, HT29, MDA-MB-231, MCF-7, and SKBR3). The IC_50_ range was estimated at 5.58 to 11.13 μM shows that compound d1 had remarkable anticancer activity on HeLa cell lines. Besides, it was discovered that d1 incited the mitochondrial apoptotic pathway by controlling Bax and Bcl-2 transcripts by expanding the Caspase 3 activation. We depicted the *in-vivo* effects of tumor advancement and the antiangiogenic activity of d1 in the EAC animal model. Tumor growth had inhibited and without symptoms the longevity of EAC containing mice expanded by the treatment of d1. Inhibition of nuclear transcriptional factor HIF-1α in EAC cells and finally it also inhibited phosphorylation of downstream signaling proteins such as ERK_1/2_, p38, and JNK in HeLa cells. The present investigation uncovered that d1 indicated noteworthy tumor-repressing abilities much less concentration in *in-vitro* and *in-vivo* recommended that compound d1 as the potent anticancer medication.

## INTRODUCTION

Cancer is a death-defying disease that causes a genuine medical issue around the world. In multicellular organisms, cancer is a multistage disease that is characterized by alteration in various gene expressions, prompting deregulated equalization of cell death that outcomes in the development of tumor growth [1–4]. Apoptosis is programmed cell death and the most extensively investigated mode of cell death that occurs in different pathological and physiological situations [5, 6]. Apoptosis plays an important role in the control of normal development, aging, and tissue homeostasis by facilitating the removal of unwanted, damaged, infected cells, or mutated cells [7]. Apoptosis is an energy-dependent manner in which the typical morphological changes occur; it includes cell membrane blebbing, cell shrinkage, and chromatin condensation, the formation of the cytoplasmic vacuole, and DNA fragmentation [8, 9]. The cell fragments generated by apoptosis are taken up and eliminated by neighbouring cells through phagocytosis [10]. At the molecular level, apoptosis is firmly controlled by the activation of cysteine-dependent aspartate directed protease (Caspase) cascade [11]. The mitochondrial pathway is managed by initiating favourable to apoptotic proteins BAX and Bcl2 inhibition [12, 13]. Cell stress incites supportive of apoptotic protein BAX to move to the outside of the mitochondria, where it multiplies pores in the mitochondrial membrane and grant the arrival of cytochrome C into the cytoplasm. When the cytochrome C is delivered, it enacts initiator caspase-9 and results in the cleavage of effector supportive of caspase-3, - 6, and 7 activations. The active Caspase-3 and - 7 explicitly cleaves poly (ADP-ribose) polymerase (PARP) that will prompt the nucleosomal DNA discontinuity Cleavage of a definite number of key proteins is significant for the improvement of apoptotic events [14].

Angiogenesis is the process in which the formation of new vessels occurs from pre-existing vasculature [15]. Angiogenic inhibitors are used to prevent the growth of the pre-existing blood vessel that is required for tumor development [16]. Angiogenesis inhibitors such as bevacizumab bind to the growth factors like VEGF thus affects the initiation of VEGF receptors [17]. VEGF completes its activity by binding to its receptors (VEGFR-1 like VEGFR-2) on the endothelial cells, enacts the downstream signaling pathway at long last prompting tumor vascularization [18, 19]. Binding of explicit ligands/development factors to its receptor instigates receptor dimerization and autophosphorylation, bringing about the enactment of the intracellular signaling pathway, which manages cell separations, development, relocation, and apoptosis [20–22]. Angiogenic inhibitors are utilized to prevent the development of blood vessels that are required for tumor development factors like VEGF, in this manner influences the initiation of VEGF receptors. Sorafenib and Sunitinib are other angiogenic inhibitors that tie to receptors on the outside of endothelial cells or different proteins in the downstream signaling pathway by obstructing their action [23]. Gefitinib, Erlotinib, and Brigatinib are small molecule kinase inhibitors which legitimately focuses on the EGFR. These days, scientists are consolidating against angiogenic drugs with chemotherapy drugs or different sorts of focused treatments for fighting malignant growth.

**Figure 1:**
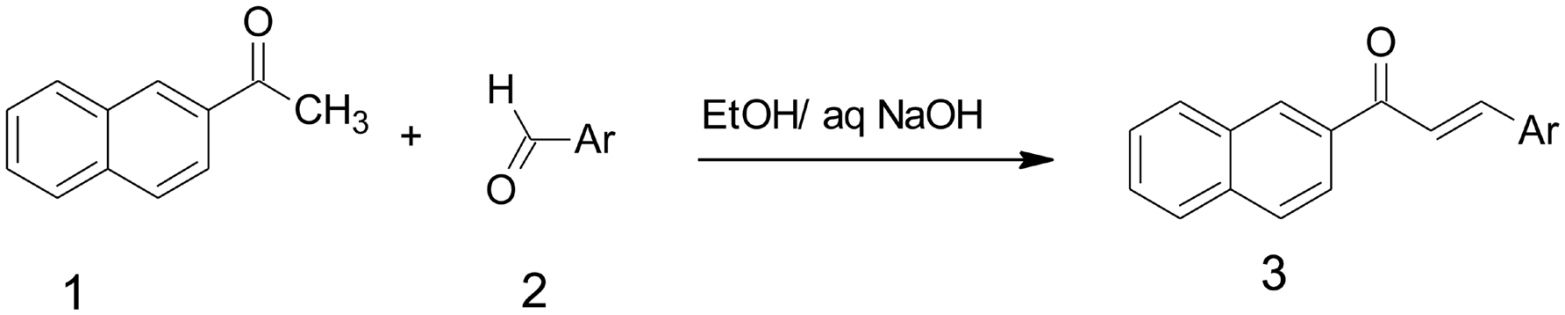
Synthesis of Naphthalene chalcone d1. For the synthesis of compound 3, 5-difluoro-4-hydroxy-benzaldehyde (0.58 mmol) d1, the compound were then stirred at ambient temperature for 4 h, an excess of ice-cold water was added, and solid products are collected by filtration and dried in the air, obtained desire product.

By the above consideration, naturally occurring compounds are extensively searched for potent anticancer medication due to their less toxic effects on human life. Chalcones are a class of natural flavonoids which comprises two aromatic rings joined by α, β-unsaturated carbonyl system. The, α β-unsaturated carbonyl framework empowers chalcones and is richly present in nature from greeneries to higher plants [24–28]. They consist of an aromatic compound with an unsaturated side chain and are effective drugs for both *in-vitro* as well as *in-vivo* animal models [29]. Through Claisen-Schmidt condensation the chalcones were synthesized which includes aldol condensation of suitable aldehyde and ketone by acid or base-catalyzed and followed by dehydration. They contain the keto-ethylenic group (–CO–CH = CH–). Both benzene rings of chalcones are completely delocalized π-electron systems and it has two-fold conjugate bonds. Chalcones bear an excellent synthon. Thus, an assortment of novel compounds with great pharmaceutical profiles can be implemented. The molecules of this classification exhibit a huge variety of biological activities, with antibacterial [30], anticancer [31], anti-inflammatory [32], antimalarial [33], and antitubercular activities [34–36]. Chalcones have electron-rich naphthyl rings that involve π-π communication; image stacking communications have made a significant part in potent inhibitors of enzymes in their active sites in the living system [37, 38]. Chalcone naphthalene is significant mostly in various human tumors. The ideal goal of the newly synthesized compound is to complete the removal of the cancer cells without damaging the normal cells using chemotherapeutics and anti-angiogenic drugs. Naphthalene bearing chalcone was carried out by base-catalyzed Claisen-Schmidt condensation of 2-acetyl naphthalene and aromatic aldehyde using ethyl alcohol as a solvent with good yield (Figure 1). The endeavour has been made in the present examination to integrate and described naphthalene chalcone (d1) by the Claisen–Schmidt condensation. In the aqueous sodium hydroxide solution for preparation of equimolar quantities of 2-acetylnaphthalene and their appropriate aldehyde were used (Figure 1).

## RESULTS

### Synthesis of chalcone of the Naphthalene (d1)

Naphthalene chalcone (d1) can be synthesized from the Claisen–Schmidt condensation reactions between equimolar quantities of 2-acetylnaphthalene using appropriate aldehyde in the presence of aqueous sodium hydroxide solution, as shown in Figure 1.

For the synthesis of compound 3, 5-difluoro-4-hydroxy-benzaldehyde (0.58 mmol) d1, the compound were then stirred at ambient temperature for 4 h, an excess of ice-cold water was added, and solid products are collected by filtration and dried in the air, obtained desire product and the compound d1 details were shown in Table 1.

**Table 1:** Details of compound d1

Compound	Molecular weight	Yield	Melting Point
d1	310.29	80.10%	160–162°C

### The cytotoxic examination in human cancer cell lines

Based on the MTT assay data, the resultant compound d1 was examined for *in-vitro* cytotoxic action in human cancer cell lines such as MDA-MB-231, MCF-7, and SKBR3 (Breast adenocarcinoma), HCT116, HT29 (Colorectal Cancer), and HeLa (cervical cancer). Reduced forms of formazan crystals were obtained from MTT- a tetrazolium salt. The viability of cells was directly measured by the intensity of the formation of formazan. IC_50_ estimation of 7.13 μM concentration reveals that the d1 shows a potent cytotoxic effect in HeLa cells. (Table 2).

**Table 2:** IC_50_ of the compound d1 in different cancer cell lines

Compound	HeLa	HCT116	HT29	MDA-MB-231	SKBR3	MCF7	Chang liver (Normal cell lines)
d1	7.13 ± 0.8	18.45 ± 0.52	21.23 ± 1.2	27.23 ± 0.45	29.89 ± 0.8	32.12 ± 1.3	> 50
Cisplatin	3.9 ± 0.6	2.8 ± 0.89	1.87 ± 1.2	3.4 ± 0.9	2.4 ± 0.24	3.2 ± 1.3	> 25

IC_50_ of all the compounds with all the cell lines was determined by 0.7–200 μM concentration in graph pad prism 5.0.

### Geimsa staining

**Figure 2:**
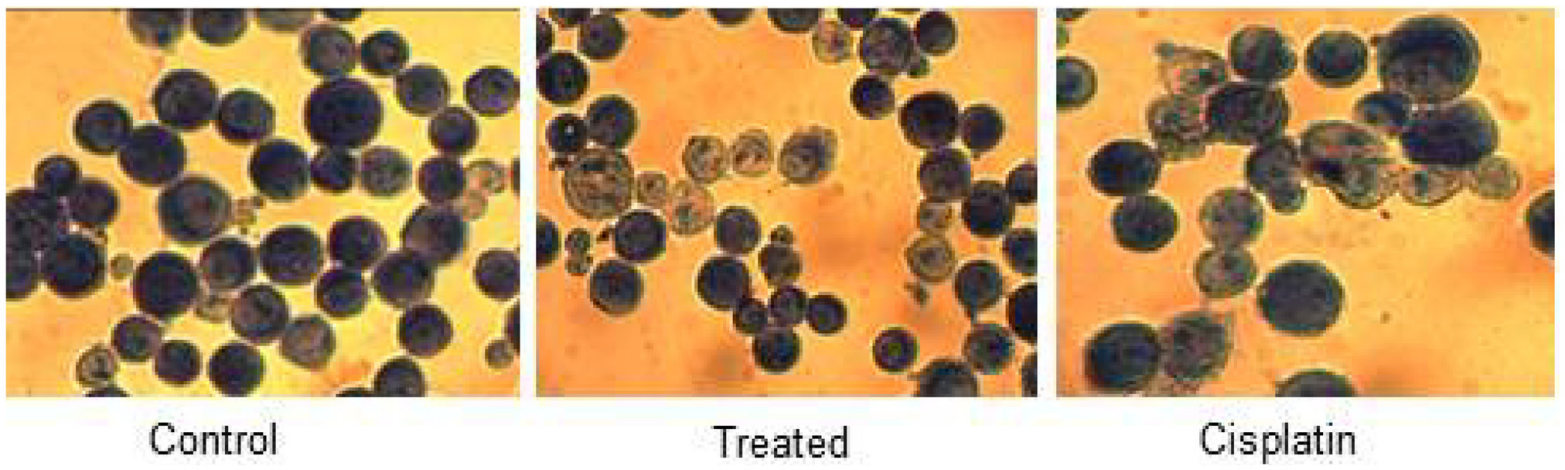
HeLa control, treated and cisplatin treated cells in d1 were fixed and stained with Giemsa and visualized under light magnifying lens and captured. It demonstrated cell shrinkage and development of apoptotic bodies in treated cells when compared with control.

Microscopic assessment of cell morphology of HeLa cells uncovered the characteristics of apoptotic hallmarks such as cell shrinkage, the arrangement of little blebs, and apoptotic bodies in d1 treated cells (Figure 2). The nuclear condensation and formation of apoptosis bodies indicate that d1 induce apoptosis in treated cells when compared to that of control cells which showed intact nuclear architecture.

### Analysis of BAX and Bcl2 gene expression

**Figure 3:**
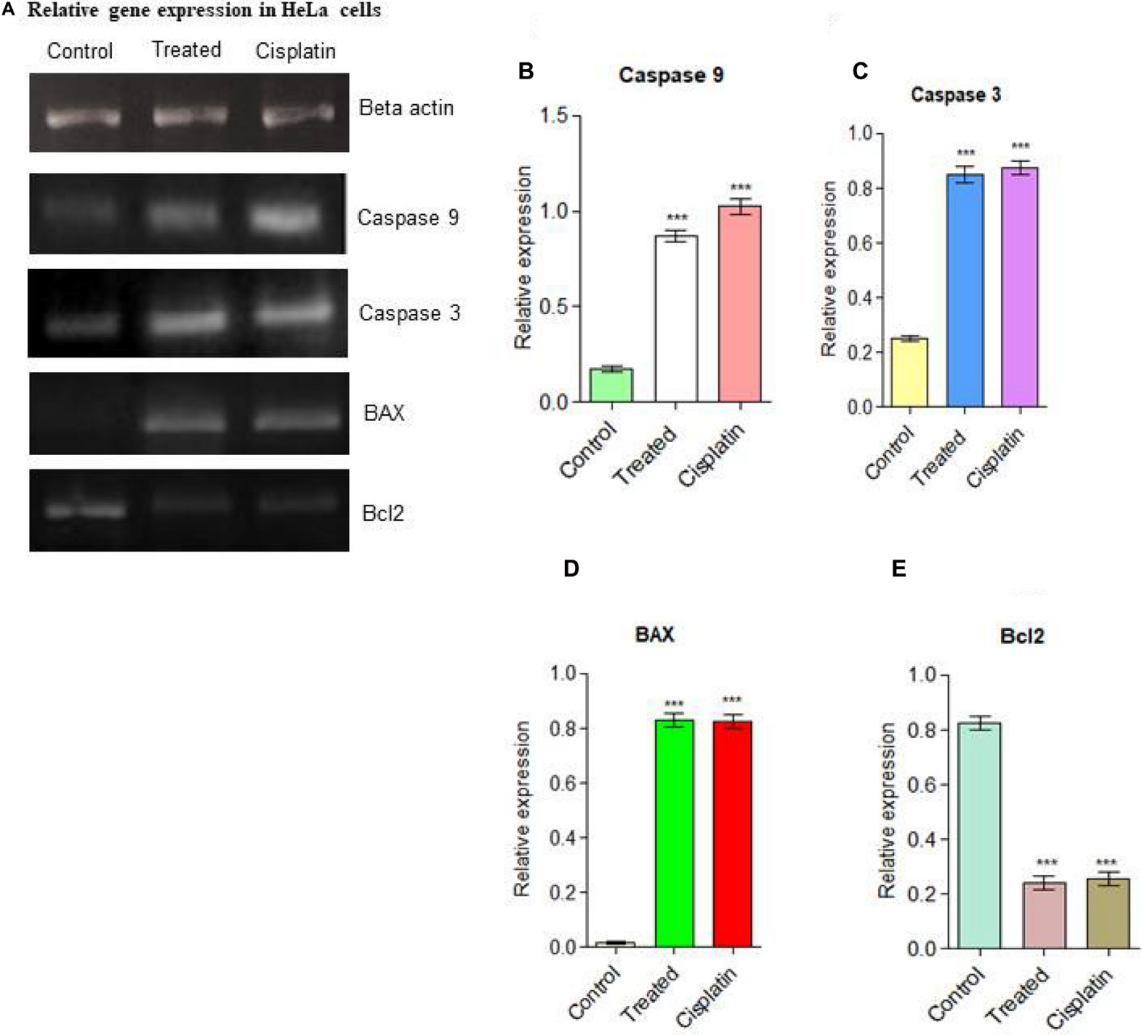
(**A**) Relative gene expressions in RT-PCR analysis for apoptotic pathway initiated by Caspase 9, Caspase 3, the up-regulation of BAX and down-regulation of Bcl2 was examined in HeLa cells. (**B**) Caspase 9, (**C**) Caspase 3, (**D**) BAX up-regulation (**E**) Down-regulation of Bcl2. Statistical significance was expressed as ^***^ *P* < 0.0001 by using one-way ANOVA.

The apoptotic signaling pathway in which compound d1 activates apoptosis via down-regulation of Bcl2 and up-regulation of BAX in HeLa cells was depicted by measuring the changes in expression levels of apoptotic proteins like BAX and Bcl2 [39, 40]. The Bcl2 family proteins play a significant role in the process of apoptosis and act both as activators or inhibitors of apoptosis. Of this, the anti-apoptotic protein Bcl2 and pro-apoptotic protein BAX plays an important role in cell death [41]. Induction of mitochondrial apoptotic pathways by d1 was investigated by evaluating the level of Bcl2 and BAX expressions, which is a crucial regulator of the mitochondrial apoptotic pathway. In the present examination compound, d1 treated HeLa cells exhibit a massive increase in the level of pro-apoptotic protein BAX expression (Figure 3A–3E). So our results suggested that compound d1 may disturb the ratio of BAX and Bcl2 levels in the HeLa cells. Compounds d1, activates apoptosis process by Increased level of BAX expression strongly indicates the involvement of intrinsic apoptotic signaling pathway in HeLa cells.

### Up-regulation of BAX by d1 leads to the PARP cleavage in HeLa cells in Western blot analysis

**Figure 4:**
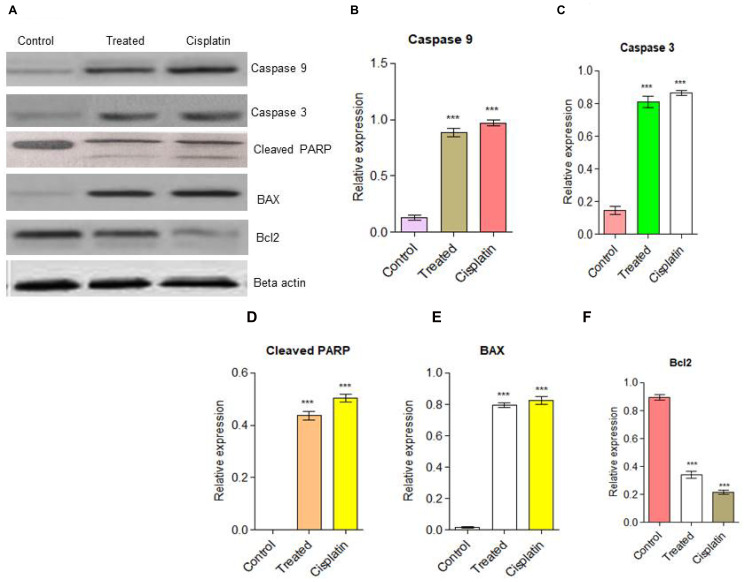
(**A**) Western blot analysis for apoptotic pathway initiated by Caspase 9, Caspase 3 and later execution pathway activates. The up-regulation of BAX and down-regulation of Bcl2 was examined in HeLa cells. (**B**) Caspase 9, (**C**) Caspase 3, (**D**) PARP cleavage (**E**) BAX up-regulation (**F**) Down-regulation of Bcl2. Statistical significance was expressed as ^***^ *P* < 0.0001 by using one-way ANOVA.

The western blot results clearly revealed that activation of Caspase 9, Caspase 3, and PARP-cleavage in d1 treated cells, whereas in untreated cells was intact. We also found that an increase in the level of BAX leads to the activation of Caspase 9 is an indication of the activated intrinsic apoptotic pathway [42]. Activated Caspase 9 activates the Caspase 3 protein, subsequently; it helps to the cleavage of PARP protein. From the above investigations, compound d1 was found to be effectively regulating the expression of BAX, Caspase 9, and PARP cleavage. This data suggested that compound d1 provides strong suggestions that the compound d1 induces apoptosis in HeLa cells (Figure 4A–4F).

### The effect of d1 on MAP kinase downstream signaling cascade

**Figure 5:**
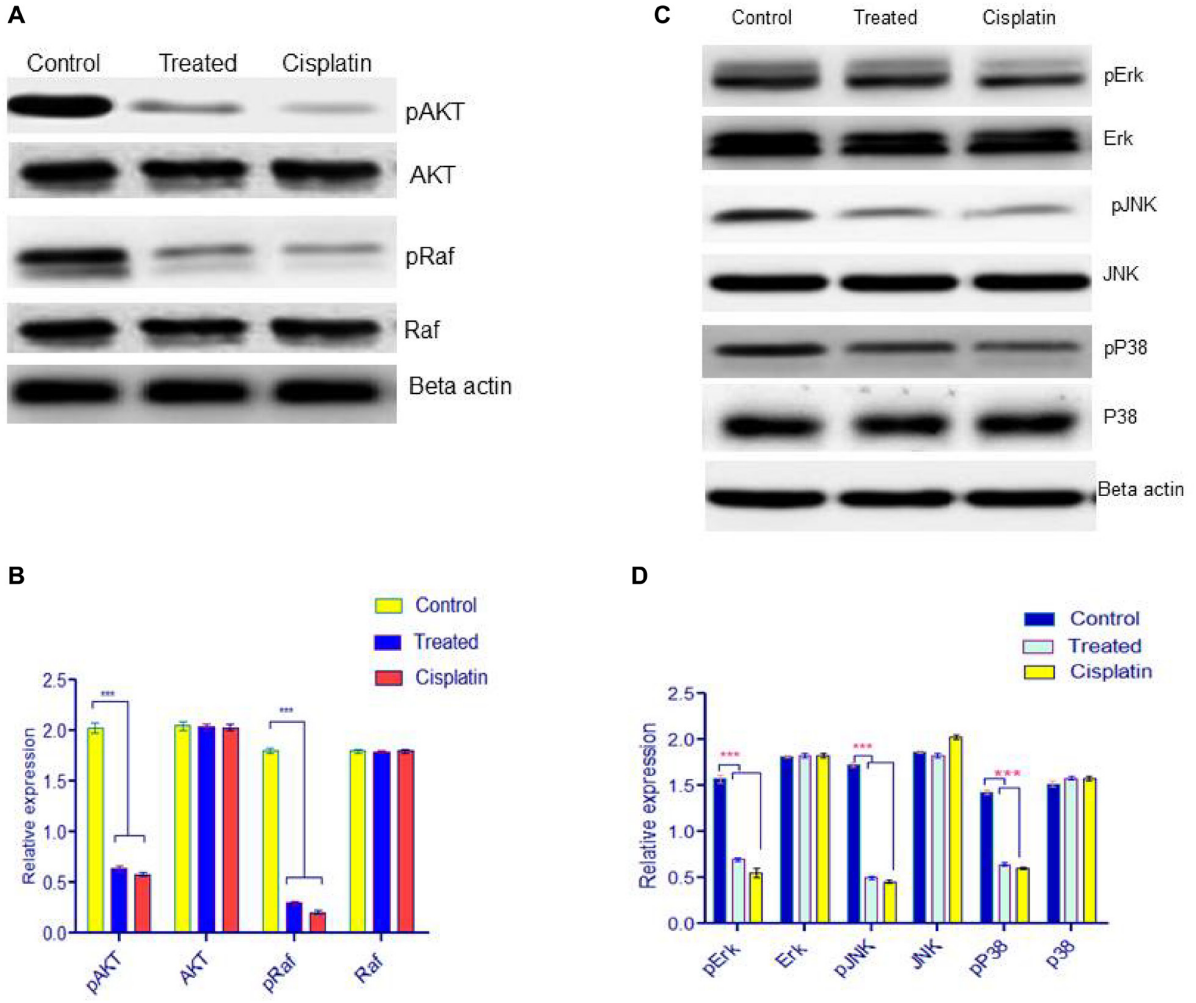
(**A**) Western blot analysis for downstream signaling pathway was examined by pAKT, AKT, pRaf, Raf. (**B**) Statistical significance was expressed as ^***^ *P* < 0.0001 by using Two-way ANOVA. (**C**) HeLa cells were treated with d1 and were resolved on 12.5% SDS-PAGE. Western blot analysis was performed using antibody for ERK, p38, JNK and the results show inhibition of phosphorylation p-ERK, p-p38, pJNK in treated with d1, cisplatin treated and control. Actin was used as loading control. (**D**) Statistical significance was expressed as ^***^ *P* < 0.0001 by using Two-way ANOVA.

Activation of MAPK is important for angiogenesis. In order to evaluate the effects of d1 treatment on the MAPK signaling pathway, following phosphorylation of AKT, Raf, p38, and pJNK were evaluated using western blot analysis. The results demonstrated that d1 inhibited the phosphorylation of AKT, Raf, p38, and pJNK. The result indicates that d1 inhibits the activation of the MAPK signaling pathway in HeLa cells (Figure 5A–5D).

### Inhibition of angiogenesis in *in ova* CAM model

**Figure 6:**
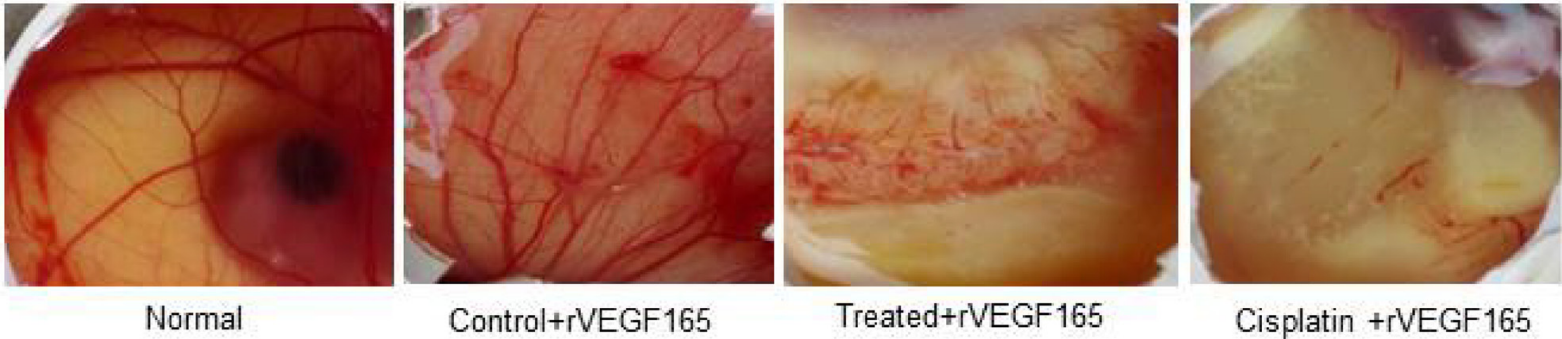
*In-vivo* CAM photos appearing decreased degree of angiogenesis in d1 and cisplatin treated eggs. rVEGF_165_ initiated angiogenesis in control and we have used rVEGF_165_ for all the eggs for determination of antiangiogenic effect in d1 as well as cisplatin.

The anti-angiogenic activity of compound d1 was examined using peritoneal angiogenesis and CAM assay. In the CAM model, a potent angiogenic stimulator recombinant VEGF_165_ (rVEGF_165_) was used to activates the development of new blood vessels. The d1 found to the most active anti-angiogenic agent. After treatment with d1, diverging of blood vessels below the discs loaded with d1 was dramatically reduced, large pre-existing vessels were also reduced and the area below the disc was found to be free from the capillary network. The absence of neovasculature below the disc showed a greater anti-angiogenic effect and the results were extremely significant (Figure 6).

### The activation of anti-tumor activity by compound d1 significantly increase the survivability of treated mice

**Figure 7:**
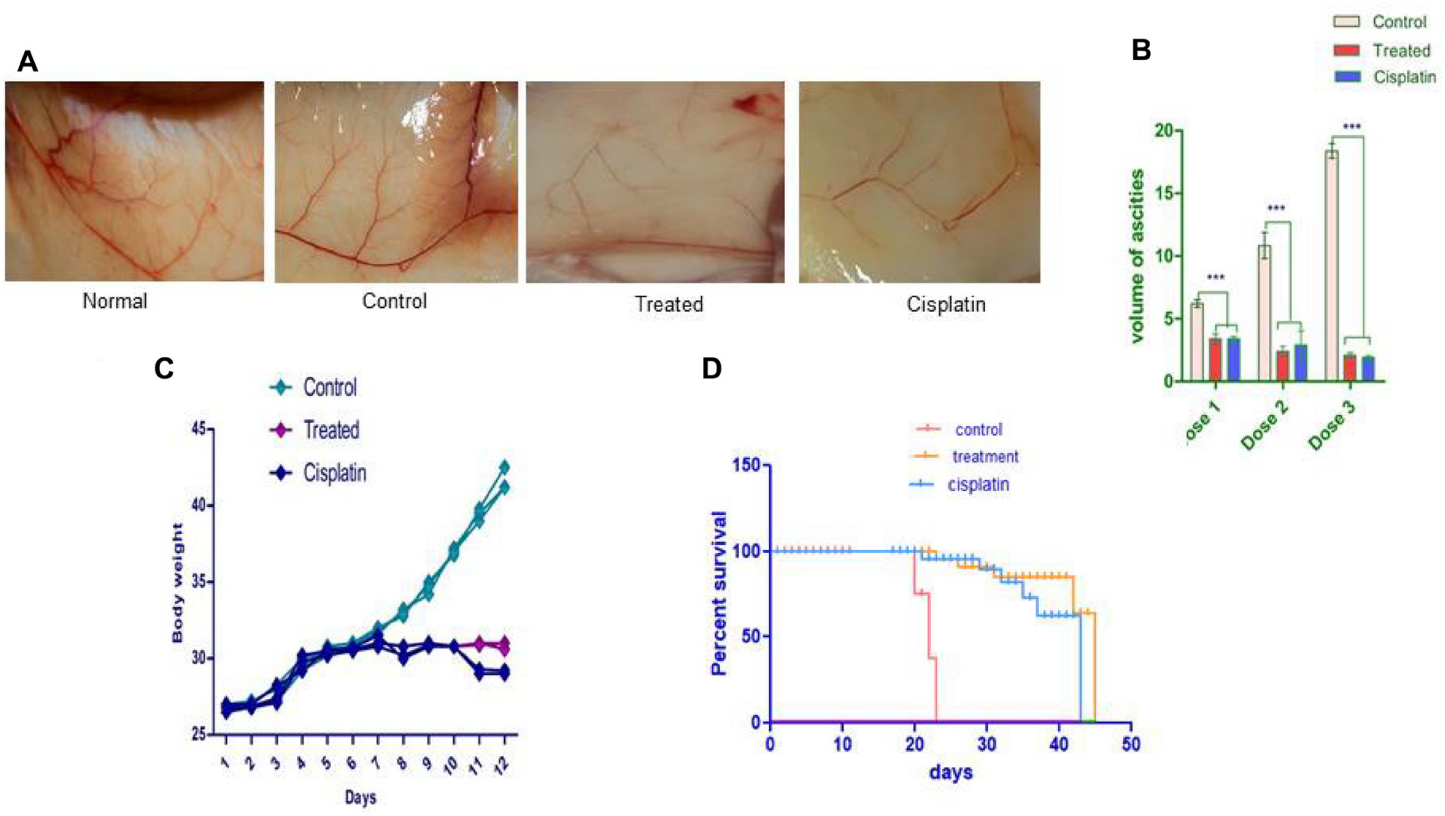
The d1 diminishes the expansion of the Ehrlich ascites carcinoma shows *in vivo*. EAC cells were cultured *in-vivo* and directed with 20 mg/kg b.w. (i.p.) of d1 of three dosages and tumor, factors were examined. (**A**) Decreases the body weight of EAC tumor-bearing mice. (**B**) The d1 diminishes the expansion of the Ehrlich Ascites Carcinoma shows *in-vivo*. EAC cells were cultured *in vivo* and directed with 20 mg/kg b.w. (i.p.) of d1 of three dosages and tumor, factors were examined. (**C**) Decreases the body weight of EAC tumor-bearing mice (**D**) the Kaplan–Meier chart bend demonstrates the all-encompassing survivability of d1 treated mice. The outcomes are the methods for (*N* = 7) judgments. Statistical significance were expressed ^***^ *P* < 0.0001.

Before conducting the *in-vivo* studies in the EAC model, toxicity studies revealed that animals treated with compound d1 up to 2000 mg/kg body were nontoxic, also there was no reduction in the bodyweight of the treated mice was observed when compared to normal mice (Figure 7A–7D). We implemented the EAC bearing animal model to examine the activation of apoptosis in compound d1. On the 6th day of tumor implantation, the d1 compound was administrated intra-peritoneal into EAC-bearing mice on every alternative day as the dose of 20 mg/kg body weight was determined by the experiment (Figure 7B). The external morphology of tumor-bearing mice treated with d1 shown that there was a significant decrease in the tumor volume when compared to EAC bearing control mice. After the three alternative treatments with d1, there was a gradual reduction in the mice’s body weight measured by detectable tumor morphology (Figure 7C). There was a dose-dependent regression in ascites volume that resulted in 80% suppression after three alternative treatments of the compound d1 while in control mice; intensification in the body weight was determined. Additionally, the *in-vivo* effect of d1 on the survivability of mice both in treated and untreated EAC-bearing mice was assessed. We detected a significant increase in the life efficacy of d1 treated mice by 47 days at the dose of 20 mg/kg body weight respectively in comparison with control (Figure 7D). Evidently, the increased percentage in the lifespan of mice treated with d1 at 20 mg/kg body weight was quite significant when compared with standard drug cisplatin.

### Histopathological studies for toxicity

The present study revealed that there was a tumor regression in d1 treated mice due to reduction or inhibition of development of EAC cells when compared to EAC-bearing control mice, increased tumor volume due to the proliferation and growth of EAC cells when compared to control mice. The d1 treated mice showed restoration of hematological parameters to the almost near-normal range. The elevated level of biochemical Parameters such as ALP, creatinine, and urea in tumor-bearing mice leads to dysfunction of the liver and kidney. After treatment with d1, the levels of liver, spleen, and kidney enzymes were restored to normal range. This result clearly suggests a significant increase in the levels of liver and renal function of EAC bearing mice treated with d1. Further, Histopathological analysis revealed that examination of tumor tissue displayed cell death in d1 treated mice when compared to control. The adverse effect of d1 treated mice displayed no reduction in body weight and hematological parameters. Section of spleen and liver also exhibited normal morphology. This shows that naphthalene chalcone d1 acts as a potent anticancer activity with no such side effects.

**Figure 8:**
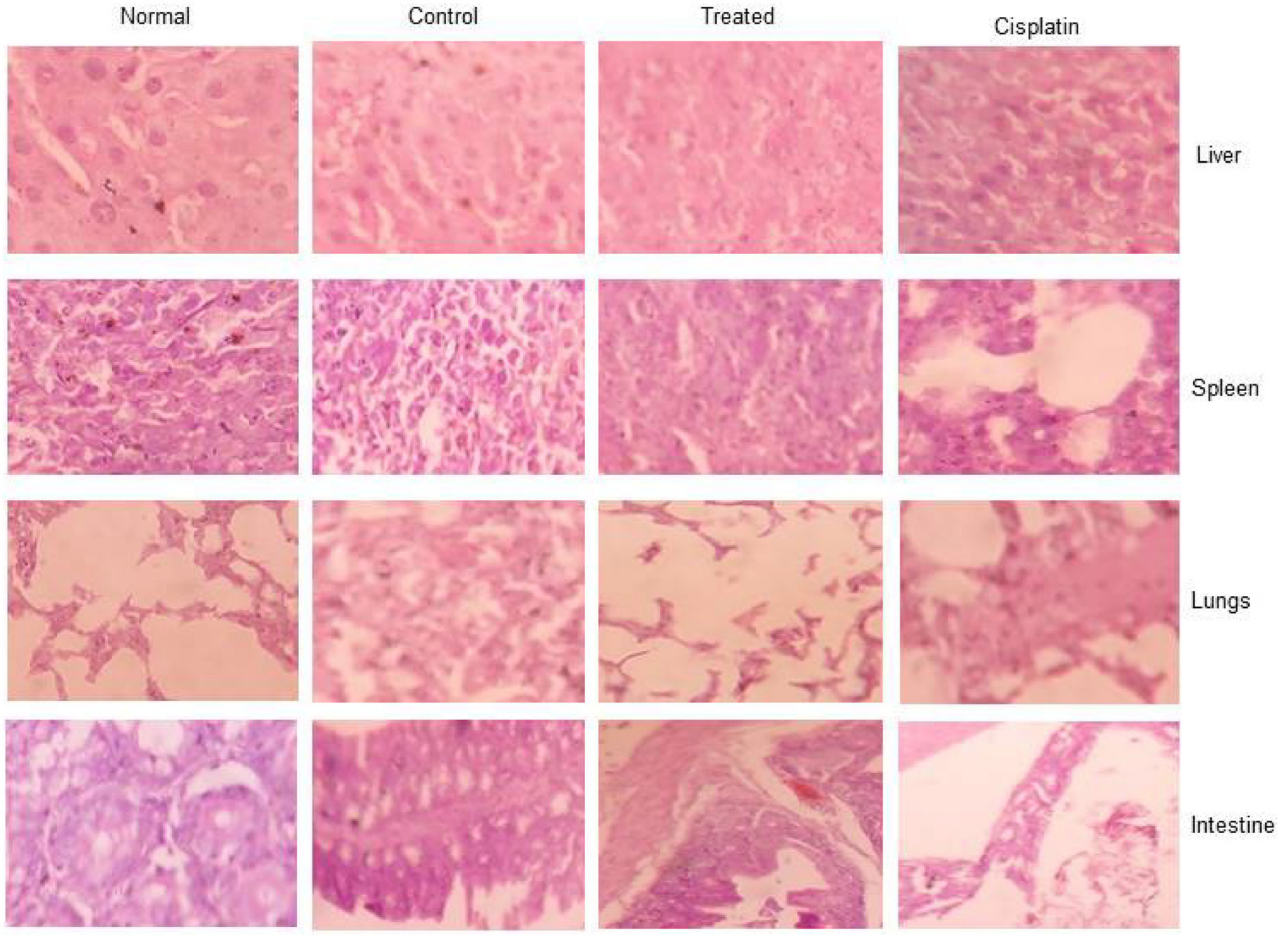
The toxicology studies for d1 in EAC animal model for liver, spleen, lungs, and intestine.

As a Histopathological evaluation, the flagrant morphology of analyzed inward organs of normal, control, untreated, EAC-bearing mice was investigated. Due to the strongly unusual developed liver, Lung, intestine, and spleen in control EAC tumors containing mice like those contracted with treated EAC mice increased to the normal state (Figure 8). Results illustrated that d1 controlled ordinary mice did not appear to have caused any toxicity. Treated EAC mice show an almost distinctive hematological profile, contrasted with control mice. Histopathological studies comprised evaluation of internal organs of the lung, liver, intestine, and spleen. Common spleen exhibits common histology with well-placed red and white pulp areas. Perceived are commonly situated lymphoid follicles admixed with splenic sinusoids and entrapped for erythrocytes. Control spleen exhibits a mixed area of passive congestion with distorted splenic sinusoids. Perceived are foci of fibrosis and increased capsular thickness with sinusoidal dilatation. Treated spleen exhibits admixed areas of congestion and fibrosis with predominantly normal splenic histology. Splenic sinusoids exhibit admixed areas of hemorrhage with normal histology. Cisplatin treated mice exhibit admired areas of mild fibrosis and hemorrhage regions with normal splenic histology. Occasional foci of entrapped RBCs are normal with sinusoidal space (Figure 8).

Common liver exhibits common hepatocytic lobular morphology with common oriented central vein and portal triad. Lobular morphology along with sinusoidal space exhibits common morphology with hepatocytes exhibiting common changes. Control liver exhibits admixed regions of disrupted central vein and portal triad with mild to moderate distortion of the hepatic lobule. Perceived regions of mild fibrosis and degeneration involving little hepatocytes with congestive regions intervene. Treated liver exhibits mainly common morphology. Cisplatin treated mice exhibit common hepatocytic histology with little areas of congestion. Apparent commonly oriented central vein and portal triad without degenerative zones. Spleen, normal: shows normal histology of spleen with well-placed white and red pulp areas. Seen are normally situated lymphoid follicles admixed with splenic sinusoids and entrapped few erythrocytes. Spleen, Control: shows distorted splenic sinusoids with admixed areas of passive congestion. Seen are foci of fibrosis and increased capsular thickness with sinusoidal dilatation. Spleen, Treated: shows predominantly normal splenic histology with admixed areas of congestion and fibrosis. Splenic sinusoids show normal histology with admixed areas of hemorrhage. Spleen, cisplatin: shows normal splenic histology with admixed areas of mild fibrosis and hemorrhagic areas. Sinusoidal space is normal with occasional foci of entrapped RBCs. (Figure 7).

Lung, normal: shows normal histology of lung with alveoli, terminal bronchiole, and parenchyma showing unremarkable changes. Seen are normally oriented and well-delineated alveoli and terminal air spaces with no distortion of architecture. Lung, Control: shows mild to moderate distortion of alveoli and terminal bronchiole with the admixed lining showing hyperplastic changes. Seen are distorted alveolar walls with features of congestion and fibrosis intervening. Lung, treated: shows predominantly normal lung histology with occasional areas showing mild alveolar wall distortion. Intervening parenchyma shows hemorrhage and mild chronic inflammatory features. Lung, cisplatin: shows normal lung histology with admixed areas of chronic inflammation and fibrosis, No alveolar distortion seen (Figure 8).

Intestine control; Sections studied show mildly distorted mucosal glands with few showing dispersed patterns. Seen are few glands showing hyperplastic to mucosal ulceration features. Intestine normal; Sections Studied show normal intestinal mucosa with mucosal glands closely arranged and cells showing normal basal polarity, no mucosal ulceration/erosion/degeneration seen. Intestine treated: Sections studied show predominantly normal intestinal mucosa with closely bound mucosal glands showing mild hyperplastic changes. No ulceration/erosion/degeneration seen.

Intestine cisplatin: Sections studied show predominantly normal intestinal mucosa with glands showing normal polarity. No mucosal ulceration/erosion/degeneration seen.

### Inhibition of nuclear translocation of hypoxia inducible factor by compound d1

**Figure 9:**
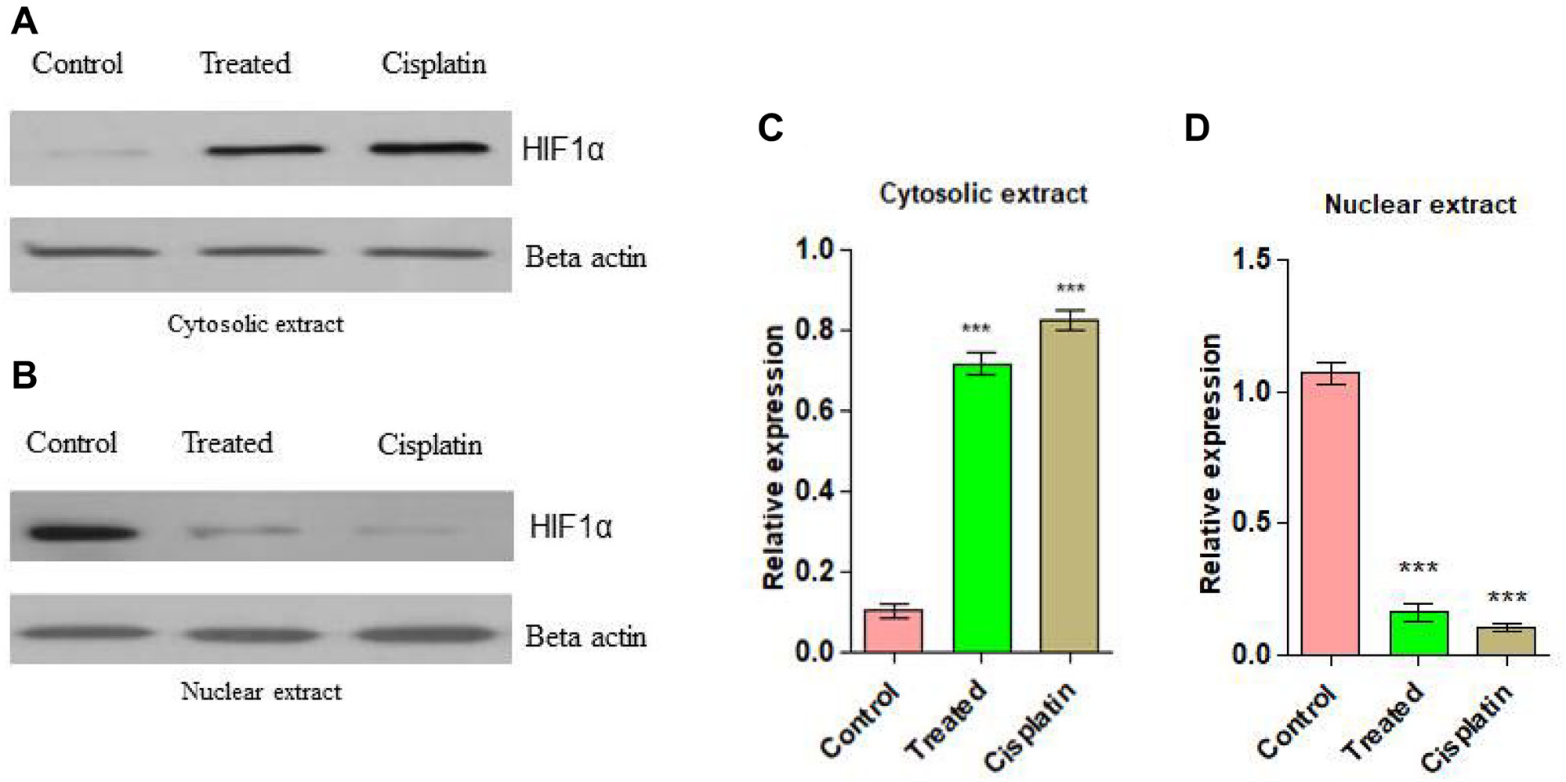
(**A**) Western blots analysis for anti-angiogenic action on d1 in Cytosolic extract from EAC cells. (**B**) Anti-angiogenic action on d1 in nuclear extract. (**C**) Graphical representation of cytosolic expression level in EAC cells. (**D**) Graphical representation of cytosolic expression level in EAC cells. Statistical significance was expressed as ^***^ *P* < 0.0001 by using one-way ANOVA.

In order to examine the essential mechanism of anti-angiogenesis of d1 in the nuclear translocation of HIF-1α was studied. HIF-1α is a transcriptional factor where its expression is well-known to be answerable for VEGF gene expression. It was perceived that in *in-vivo* treatment of d1 showed less expression of HIF-1α protein level in nuclear extract than in the cytosolic protein extract, whereas in control cells increased expression level of HIF-1α in the nuclear protein extract was observed. This suggested that a cell treated with d1 inhibits the HIF-1α translocation into the nucleus (Figure 9A–9D).

## DISCUSSION

In the present study, compound d1 was shown to induce cytotoxicity in cancer cells in *in-vitro* by activating the mitochondrial apoptotic pathway. We have focused to depict the apoptotic effect of the d1 on HeLa cells. Cell death is accompanied by the activation of a class of caspases and extensive biochemical and morphological changes in the cells. Cell shrinkage, membrane blebbing, chromatin condensation, and formation of apoptotic bodies are morphological features of apoptosis. To examine whether the cytotoxic effects induced by the compound d1 in HeLa cells involve typical morphological changes, cell were examined with Giemsa staining. The d1 and cisplatin-treated HeLa cells were stained with Giemsa exhibited membrane blebbing, the formation of apoptotic bodies, and chromatin condensation. Activation of proteolytic enzymes such as cysteine protease might be correlated with the major apoptotic signaling cascade.

Apoptosis of the Caspase 3 execution pathway is an important sign for apoptosis and can be used in the cellular assay to identify the death cascade. Caspases are synthesized as enzymatically inert zymogens and are usually activated by proteolytic cleavage. The response is both concentrated specific and time-dependent manner, proposing that many pathways play a significant role in the activation of Caspase 3. Caspase 3 is mainly responsible for the most apoptotic events and its cause degradation or death of the cells to evaluate the d1 on treated cells, a significant amount of cells were encouraged to undergo apoptosis via Caspase 3 activation and the effects were examined by RT-PCR and western blot analysis.

The intrinsic apoptotic pathway is controlled and regulated by the Bcl2 family proteins. The Bcl2 family proteins are known to be an important role in apoptotic responses. The initiation of apoptotic depends on the balance between pro-apoptotic and anti-apoptotic proteins that leads to the activation of cell cycle arrest and finally cell will undergo for death. In some cancer types, the anti-apoptotic protein Bcl2 plays a significant part in the formation of cancer progression [43]. The overexpression of Bcl2 in many cancer cells may inhibit the pro-apoptotic signals which allow cancer cells to survive under stress conditions. The anti-apoptotic protein Bcl2 prevents apoptosis either by preventing the release of mitochondrial apoptotic factors such as cytochrome C and AIF into the cytoplasm or by sequestering the perform of death-inducing cysteine proteases called Caspase. Cells become sensitive to apoptosis when the level of pro-apoptotic protein BAX increases. A high level of pro-apoptotic Bcl2 family protein BAX sensitizes the cells for apoptosis. The pro-apoptotic protein BAX triggers the activation of Caspase and release of the mitochondrial apoptotic pathway by the release of cytochrome C and Apaf-1 into the cytoplasm through transition pores, thereby leading to the activation of Caspase. In the present examination, d1 and cisplatin suppressed Bcl2 mRNA expression and increased the expression level of BAX. Immunoblotting analysis of HeLa cells was treated with compound d1 and cisplatin displayed active Caspase 9 when compared to control. Activation of Caspase 3 produces typical cellular morphology features of cell death. The activated Caspase 3 cleaves its substrate PARP family of cysteine proteases involved in DNA damage repairing. Cleavage of PARP leads to the destruction of DNA within the nucleus which is the hallmark feature of apoptosis. The result from western blot analysis of whole protein extract of HeLa cells treated with compound d1 showed activation of Caspase 9 by apaf-1 and Cytochrome C. These results show that compound d1 significantly activates the Caspase 3 in HeLa cells. Further results showed that activated Caspase 3 executioner protease then cleaves PARP, which is responsible for the activation of apoptosis. The above data suggested that BAX protein plays an important role in inducing apoptosis via the intrinsic apoptotic signaling pathway in HeLa cells.

MAPK are receptor tyrosine kinases that mediate intracellular phosphorylation triggered by an array of extracellular stimuli including growth and differentiation factors, cytokines, and integrin-mediated cell attachment. An increased level of MAPKK activation in tumors compared with their corresponding non-neoplastic tissue has been reported in several human carcinomas. The AKT, RAF, ERK, JNK, and p38 are downstream MAPK signaling family members, also play crucial roles in cell proliferation and apoptosis. An extracellular signal-regulated kinase (ERK) regulates the intracellular pathway in response to growth and survival-promoting stimuli. Both JNK and p38 are activated by extracellular stress. Some evidence suggested that activation of p38 and JNK MAPK cascades have jointly inducing anti-apoptotic effects. So ERK is a propitious target for developing new anti-angiogenic drugs. Previous reports suggest that stimulation of VEGFR may lead to the activation of the downstream signaling member ERK, which is involved in cellular proliferation. Recent studies revealed that VEGF also activates the p38 MAPK signaling pathway. ERK can activate JNK kinases when endothelial cells are activated by growth factors such as VEGF and both MAPK mediates the proliferation of the cell. JNK pathway has been involved in both apoptosis and survival signaling pathways.

Tumor angiogenesis is one of the important targets for cancer therapy. It is very important to understand the signaling pathways that control the angiogenesis, which will assist in the identification of therapeutic drugs and the design of effective anti-angiogenic therapies. Tumor growth and metastasis depend on its vascular network. The increased vascular network increases tumor growth. Thus inhibiting tumor angiogenesis limits tumor volume and inhibits the metastatic ability of the tumors. In the present investigation, we examined the mechanism of action of d1 on the EAC tumor model indicating their anti-angiogenic efficacy. *In-vivo* studies after treatment revealed that compound d1 showed a decrease in the ascites volume and body weight of treated mice and was accessed by regular examination of the bodyweight of the mice. There was a dose-dependent manner in the ascites liquid that resulted in 80% diminishing after three alternative doses of the compound the d1. Although, in EAC-bearing control mice an enormous amount of ascites volume was increased in the body weight was observed. Further, the inhibitory effect of compound d1 on EAC cells in *in-vivo* was studied with ascites secretion and the total number of cells in treated and control mice. Ehrlich Ascites is a liquid accumulation in the area of the peritoneal cavity, which serves as a nutritional source for tumor cells for their development. Hence, a diminishing in the volume of ascites liquid leads to a decrease in ascites volume. Hence, our results demonstrated that there was a decrease in the volume of ascites liquid in compound d1 treated mice when compared to control mice. A decrease in the tumor burden is associated with the volume of ascites. These studies suggested that compound d1 was able to suppress tumor growth. VEGF plays an important role in promoting endothelial cells proliferation and invasion, increased vascular permeability, stoma degradation, and the formation of extravascular fibrin substrate for endothelial and tumor cell proliferation. VEGF is a potent vascular permeability factor that plays a pivotal role in the accumulation of ascites. It also plays an important role in the survival factor for endothelial cells by suppresses apoptosis and enhancing the formation of the newly formed blood vessels. Thus, inhibition of ascites fluid formation is possibly due to the inhibition of VEGF secretion. Angiogenesis was observable in the inner peritoneal cavity of tumor-bearing mice. Our results exhibited substantial inhibition of pre-existing blood vessel formation in the peritoneal lining of d1 treated mice, which may be due to the accumulation of angiogenic stimulating factors. The formation of pre-exiting blood vessels is due to the involvement of growth factor-like VEGF [44–47]. EAC bearing mice treated with d1 displayed a significant declination of peritoneal angiogenesis indicating that the inhibition of angiogenic factors and thereby avoiding the formation of pre-existing blood vessels. Additionally, the d1 treated EAC-bearing mice and untreated mice were examined for angiogenesis. Therefore, the anti-angiogenic effect of the d1 was able to delay the growth of Ehrlich ascites in the animal model.

Assessment of angiogenic activators is an important process for which *in-vivo* chick embryo chorioallantoic membrane serves as a suitable experimental model to correlate the process of angiogenesis. *In-vivo* anti-angiogenic effect of d1 on the chorioallantoic membrane, the model displayed avascular zone formation not only at the place of the outline of compound d1 but also in a major portion around the disc when compared with the comprehensive angiogenesis observed in the normal chorioallantoic membrane model.

The angiogenesis is frequently activated by hypoxic conditions and it is familiar that the microenvironment of the EAC animal model with cells in *in-vivo* is hypoxic and increased the expression of HIF-1α is a transcriptional factor which is involved in the activation of VEGF gene expression [48–51]. There are few antitumor agents that target HIF-1α in ascites tumor cells in *in-vivo* have been reported [52]. It evident that compound d1 has the possibility to convert the anti-angiogenic mediator to target HIF-1α. The present investigation suggested that the compound d1 might influence angiogenesis by down-regulation of the expression of nuclear HIF-1α while increasing its abundance in the cytosolic extract of EAC cells. It also shows that compound d1 suppresses the activation of VEGF in EAC cells due to the inhibitory activity of HIF-1α.

In conclusion, the present study demonstrated that compound d1 is a very potent anti-angiogenic compound that inhibits the growth of EAC cells *in-vivo*. These inhibitory effects may be related to the suppression of transcriptional factor HIF-1α nuclear translocation. HIF-1α is accountable for inhibition of hypoxic up-regulation of VEGF gene expression, resulting in decreased ascites volume and thereby inhibiting the tumor growth which is angiogenic reliant and also inhibiting the phosphorylation of downstream signaling components such as ERK, p38, and JNK of the MAPK pathway. It is also evident that inhibition of the downstream signaling pathway was due to the binding of compound d1 to VEGFR, preventing the binding of other growth factors. Such active compound d1 prove to be potential anti-angiogenic drugs that could be further developed and translated into a therapeutic regime for the treatment of various human cancers.

## MATERIALS AND METHODS

All the chemicals such as 2-acetyl naphthalene, o-hydroxy benzaldehyde, bromobenzene were purchased from CDH chemical AR grade and used as provided. The solvents, for example, benzene, ethyl acetic acid derivation, dichloromethane, dimethyl sulfoxide, petroleum ether, diethyl ether were of AR grade and utilized as such moving forward without any more filtration, Absolute Ethanol was bought from Avra synthetic compound and utilized as a dissolvable.

### Experimental section

TLC was used to monitor the reactions carried on silica gel glass plates. TLC visualization was done by iodine indicator or U.V light. VNMRS-400 “Agilent -NMR” spectrometer was used to record the ^1^H NMR data. From the internal TMS standard, the values of the chemical shifts in ppm downfield were reported. Tetramethylsilane (TMS) was used as an internal standard using the DMSO solvent.

### Synthesis of 3-(3,5-difluoro-4-hydroxyphenyl)-1-(naphthalen-2-yl] prop-2-en-1-one (d1)

Red powder, Yield 80.10%, MP 160–162^°^C,1H NMR (400 MHz, DMSO- d6): δ (ppm) 8.76 (s, 1H), 8.10–8.80 (t, J = 6, 7.6 Hz, 2H), 7.98–7.95(d, J = 5.8 Hz, 2H), 7.60–7.57 (t, J = 5.2, 8.4 Hz, 3H), 7.45–7.41 (d, J = 14.8Hz, 1H), 7.23–7.20 (d, J = 11.2 Hz, 2H).13C NMR (100 MHz, DMSO- d6) 157.553, 156.054, 155.343, 146.644, 136.931, 134.939, 132.909, 129.797, 129.313, 128. 362, 128.327, 128.014, 127.027, 124.885, 112.886, 111.575, 109.113.MS (*ESI*) m/z: 310.29 (*M*+).

### Cancer cell lines, chemicals, and antibodies details

The human malignant growth cancer cell lines MDA-MB-231, MCF-7, Skbr3 (breast cancer), HeLa (cervical cancer), HT-29, HCT 116 (colorectal adenocarcinoma) and normal cell line chang liver are procured from the National Centre for Cell Science, Pune, India. The cells were developed in Dulbecco’s Modified Eagle Medium (DMEM) medium enhanced with 10% inactivated Fetal Bovine Serum (FBS), 100 μg of streptomycin/ml, 100 U/ml of penicillin and incubated at 37 ± 2°C with 5% CO_2_. MTT dye (3-(4, 5-dimethylthiazol-2-yl) - 2, 5-diphenyl tetrazolium bromide) was acquired from Sigma Aldrich (Mumbai, India). All the experimental reagents and solvents were purchased from sigma Aldrich chemicals Pvt Ltd. Recombinant VEGF_165_ (Cell signalling technology), P-p44/42 MAPK, P-SAPK/JNK (G9) and JNK antibodies were purchased from cell signaling technology, USA. HRP-conjugated mouse secondary antibody and HRP-conjugated goat anti-rabbit secondary antibodies were purchased from cell signalling technology. Actin antibody was obtained from Abcam, USA.

### MTT cell viability assay

Cytotoxic activity of chalcones examined by applying MTT assay [53]. In 100 μl DMEM (1× 10^4^ cells/well) had seeded. 10% FBS was added in all well of 96-well micro-culture plates and incubated at 37^o^C in a CO_2_ followed by 48 h of incubation. 10 μl MTT (3-(4, 5-dimethylthiazol-2-yl)-2, 5 diphenyl tetrazolium bromide) (5 mg/mL) were added to all wells and the plates further incubated for 4 h. The supernatant from each well in 100 μl of DMSO, the crystals of formazan were taken up and absorbance was recorded at 540 nm.

### Giemsa staining

Giemsa staining was performed and visualized under a light microscope (Leitz-DIAPLAN). Both untreated, cisplatin-treated, and d1 treated cells of HeLa were collected, fixed in methanol: acidic acid (3:1) smeared on a glass slide and air-dried in a humidifier chamber. The cells were washed with PBS and stained with Giemsa. These cells were washed with PBS and visualized under a light microscope respectively [54].

### RT-PCR

It was performed to analyse β-Actin RNA articulation, BAX, Bcl2, Caspase 3, and Caspase 9. As per the manufacturer’s instruction, the cDNA synthesized from 2 μg of RNA utilizing a verso cDNA synthesis kit accompanied by oligo dT primer. Total reaction volume to 20 μl and carry out the synthesis of cDNA for 1 h at 42^°^C, pursued by R.T inactivation for 5 min at 85^°^C. Samples of resultant products were examined in 1.5% agarose gel [55].

### Western blotting

HeLa cells treated with d1 and untreated cells were collected and lysed for 1 h at 4^°^C in lysis buffer [20 mM Tris PH 7.5, 2 mM EDTA, 3 mM EGTA, 2 mM Dithiothreitol (DTT), 250 mM sucrose, 0.1 mM phenylmethylsulfonyl fluoride, 1% triton × –100] containing a protease inhibitor cocktail. Protein (50 μg) was resolved on 12.5% SDS polyacrylamide gel and transferred on to PVDF membrane. For immunoblotting, anti-antibodies were used followed by the addition of HRP-conjugated specific secondary antibody. Beta Actin was used as an internal loading control. The protein bands were developed and visualized by enhanced chemiluminescence [56].

### *In-vivo* studies

#### Animal and ethical statement

BALB/c female mice named Swiss albino, which weighed about 28 ± 1.5 g and 6–8 weeks old. They were housed under standard conditions. Water and animal food were given to mice throughout the analysis. Mice kept with proper ventilation at room temperature of 22^°^C ± 2^°^C for 12 h day/night cycle. All mice experiments were performed in Bharathi College of Pharmacy, Bharathi Nagara, Mandya District, India with the assistance from Institutional Animal Ethical Committee (IAEC) (*Approval No: BCP/IAEC/EXTP/05/2018*).

### *In-vivo* studies in EAC animal model

Ehrlich Ascites carcinoma (EAC) cells were kept in the *In-vivo* section. Under sterile condition tumors of EAC containing mice were dissected. In 0.9% normal saline the cell suspension was contrived from examined tumors. 5 × 10^6^ cells/mice were injected intra-peritoneal to the mice and developing ascites volume with stomach swelling. During the development period, the mice displayed astonishing increases in body weight and after 12th day implantation of mice were sacrificed due to the accumulation of asities tumor volume. During the 12th days of development, the number of cells expanded with an accumulation of 20–20.5 mL ascites fluid with broad neovascularization in the internal coating of the peritoneal wall was examined [57].

### The collection of ascites volume in the EAC animal model

Six to eight weeks old Swiss albino female mice weighing 28–30 g were housed. 5 × 10^6^ EAC cells were administrated intraperitoneally into the mice. On 6th day, compound d1 (20 mg/kg) body weight was injected and the compound d1 treatment was repeated on every alternative day till the 10th day. The bodyweight of treated and untreated mice was monitored throughout the experiment starting from the day 1st of translation. On 12th day mice were sacrificed and by creating a small hole in the stomach region the ascites liquid had collected. From ascites fluid, the EAC cells had segregated. For 10 mins the collected ascites fluid of mice was centrifuged at 1,500 rpm. The volume of ascites liquid determined after separating from cancer cells, EAC cells collected as pellets and used for further evaluation [58].

### Peritoneal angiogenesis

EAC-bearing mice were cut open at the stomach region and d1 influence on angiogenesis was known. The internal covering of the peritoneal cavity was opened. Control and treated mice peritoneal image was taken by using Nikon digital camera [59].

### In ova chorioallantoic membrane (CAM) assay

The angio-inhibitory effect of compound d1 was determined by the chorioallantoic membrane (CAM) assay as reported earlier [60]. Briefly, fertilized eggs were incubated at 37^°^C in a sterile humidified atmosphere. To check the proper development of the embryo during the incubation period, a window was opened by cracking the shell under aseptic conditions and resealed. The angiogenic stimulator, 10 ng of recombinant VEGF_165_ was used to induce angiogenesis. On the 10th day, the windows were opened, a sterile filter paper disc loaded with the compound, and rVEGF_165_ was placed on CAM, and the windows were released and incubated for two days. On the 12th day, observations were made for changes in the Microvessel density under the region of disc and photographed using Nikon digital camera.

### H and E staining analysis

As per standard convention we assembled and handled tissue of control, treated, and cisplatin-treated mice for pathological examination in the liver, lung, intestine, and the spleen [61]. The tissues were placed in paraffin wax, distinguished at 10 mm in rotational and stained with eosin and hematoxylin (Leica Bio systems). Each segment is assessed through an appended CCD camera. Toxicity of treated d1 mice organs, control, and normal mice organs was compared by images that were shot via a CCD camera.

### Statistical analysis

Statistical significance was accomplished by One-way and Two-way ANOVA and One-way ANOVA conveyed by Bonferroni’s Multiple Comparisons Test. It is used to examine correlation *p* < 0.05 was considered to specify a notable distinction for computing IC_50_ of all the compounds. Data were analyzed utilizing Graph Pad Prism 5.0 software.

## CONCLUSIONS

By Claisen-Schmidt condensation of Naphthalene bearing, chalcone had been synthesized. In addition, the dynamic job of the mitochondrial apoptotic pathway has been contemplated and was additionally affirmed by the activation of BAX, a principal apoptotic protein, and activation of caspase-3. It can likewise be presumed that d1 at a portion of 20 mg/kg body weight, ideally represses the development of EAC cells *in-vivo*. This was apparent from decreased tumor size and improved life efficacy of that examination animal. The treatment with d1 re-established the digressed hematological and biochemical boundaries to the ordinary range. It was shown to be a viable anticancer and hostile to the neoplastic specialists with less harmfulness. Antiangiogenic and pro-apoptotic activity studies had been accomplished and the result revealed that the compound had an electron-donating group that exhibits superior activity in the compound d1.

### Ethical approval

All applicable international, national, and institutional guidelines for the care and use of animals were followed. All mice experiments were performed in Bharathi College of Pharmacy, Bharathi Nagara, Mandya District, India with the assistance from Institutional Animal Ethics Committee (IAEC) (Approval No: BCP/IAEC/EXTP/05/2018).
